# Have increased deaths at home during the pandemic returned to pre-pandemic levels? An analysis of publicly available Scottish death registrations

**DOI:** 10.1093/pubmed/fdad156

**Published:** 2023-08-18

**Authors:** J Savinc, I M Atherton

**Affiliations:** Edinburgh Napier University, School of Health & Social Care, Edinburgh EH11 4BN, UK; Scottish Centre for Administrative Data Research, Edinburgh EH8 9BT, UK; Edinburgh Napier University, School of Health & Social Care, Edinburgh EH11 4BN, UK; Scottish Centre for Administrative Data Research, Edinburgh EH8 9BT, UK

**Keywords:** mortality, older people, places

## Abstract

**Background:**

Deaths at home increased in Scotland at the start of the Coronavirus disease 2019 (COVID-19) pandemic by ~35%. The majority did not involve COVID-19. This has implications for resource allocation and care at the end of life.

**Methods:**

Publicly available weekly death registrations by National Records Scotland (NRS) between 2015 and week 25 of 2023 were summarized by place of death. Linear and logistic regressions of the number and proportion of deaths at home, respectively, between 2015 and 2019, were used to estimate the expected number and proportion of deaths in the period 2020–2023 had the pandemic not happened.

**Results and conclusion:**

The number of deaths at home continues in 2023 at rates similar to the pandemic period and has not reverted to pre-pandemic levels. Had the pre-pandemic trend of growth in deaths at home continued, the number of deaths observed in 2020 would not be observed until 2025–2032. Deaths at home increased across Local Authorities but the scale of the increase varied. The impact of the increased number of deaths at home on quality of care and quality of death is not known and requires further study.

## Introduction

The number of people who died at home increased at the start of the pandemic across the UK,^1^^,^^2^ including Scotland,^3^ where they increased by ~35%. Most home deaths did not involve Coronavirus disease 2019 (COVID-19). The proportion of home deaths had been increasing slowly prior to the pandemic and increased markedly in the early stages of the pandemic.^3^^,^^4^

If it continues, the shift to deaths at home will have notable resource implications. Health and care needs increase with proximity to death,^5^ and with the majority of deaths associated with some degree of palliative care needs,^6^ the need for community health support, social care and caregiver support will increase, with early identification, signposting and support required for unpaid carers.^4^^,^^7^

Reasons for the increase are not clear: resources had to be shifted towards the increase in acute hospitalizations during the pandemic, and there was fear of end-of-life hospitalization due to perceived infection risk and loved ones not being able to visit.^8^ This paper builds on earlier work^1^^,^^3^ to investigate whether the number of home deaths has returned to earlier levels as the pandemic has receded in terms of COVID-19 cases, public health measures and excess deaths.^9^

## Methods

National records of Scotland (NRS) publish weekly deaths,^10^ including place of death grouped into ‘*hospital’, ‘care homes’, ‘home or other non-institutional settings’* and ‘*other’*. Cause of death was grouped into ‘all cause’ and ‘non-covid’ deaths. Data from 2015 to week 25 (ISO 8601) of 2023 were used for analysis. In addition, the number of deaths at home aggregated by Local Authority (LA) was used for 2015 to week 9 of 2023.

Descriptive statistics were calculated for the number of deaths in total by place, LA and year, and compared with the historic (2015–2019) range and mean. Linear and logistic regressions were computed for absolute number and proportion of home deaths between 2015 and 2019 to estimate the annual increase in home deaths prior to the pandemic and to extrapolate when we would expect pandemic levels of home death (see Supplementary file for details).

## Results

**Fig. 1:**
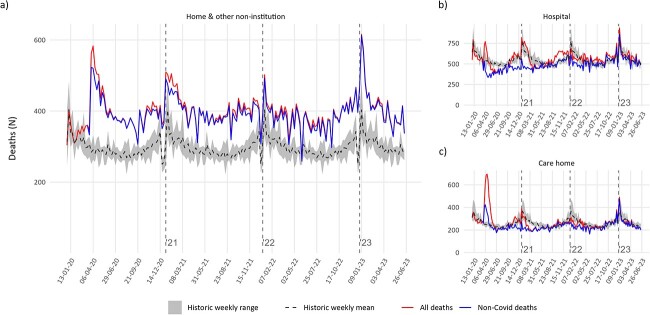
(a) Home and other non-institution, (b) hospital, (c) care home; ‘other’ place not shown due to small numbers. Data from w/c 30 December 2019 to w/c 19 June 2023 (inclusive). Note: all figures are based on date of registration. Historic mean and range for 2015–2019 throughout. Source: National Records of Scotland.

The home death increase continues into 2023 (Fig. 1a), unlike deaths in hospital and care homes which have returned to pre-pandemic levels (Fig. 1b and c). Since the first Scottish lockdown on 23 March 2020, deaths at home have consistently exceeded the weekly maxima for the 2015–2019 period. Fewer than 2% of home deaths were associated with COVID-19 in 2020–2022. Home deaths increased disproportionately compared with hospital and care home deaths (see Supplementary Fig. 1 and Supplementary Table 1) so their increase cannot be attributed solely to the overall pandemic increase in mortality.

Home deaths were increasing before the pandemic but increased drastically during the pandemic. Before the pandemic, home deaths ranged between *N*_2015_ = 14 831 (25.3% of all deaths) and *N*_2019_ = 16 166 (28.0%); during the pandemic, they increased by 128% to *N*_2020_ = 20 708 (31.9%), *N*_2021_ = 21 145 (33.2%), then reduced slightly to *N*_2022_ = 20 084 (31.9%) and as of week 25 of 2023, increased again to 32.3% of annual deaths at *N* = 10 457 (see Supplementary Table 1).

Assuming a continuing linear trend from the 2015–2019 data, we would expect an additional 338 deaths at home per year, reaching the 2020 levels of home deaths, *N* = 20 708, in the year 2032 (see Supplementary Table 2 for regression coefficients and Supplementary Table 3 for extrapolated figures). Assuming a logistic trend, the proportion of home deaths observed during the pandemic, 31.9%, would first be observed in 2025.

**Fig. 2:**
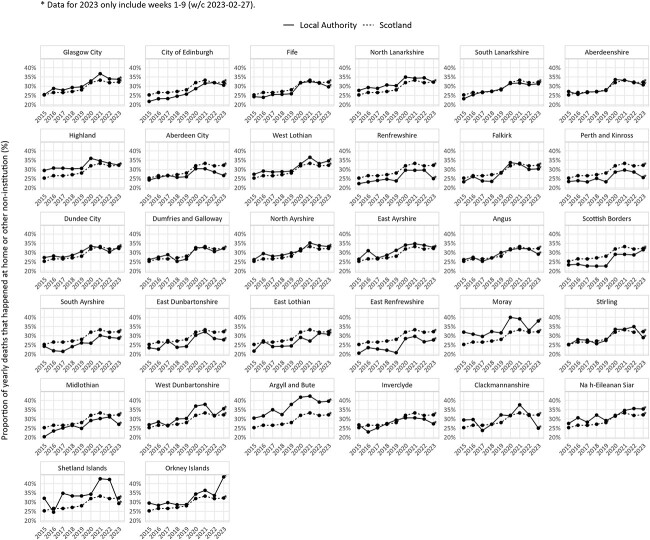
LAs are shown in order of 2020 population estimate. Data from w/c 30 December 2019 to w/c 27 February 2023 (inclusive). Note: all figures are based on date of registration. Source: National Records of Scotland.

The increase in home deaths was unequally distributed across Scotland (Fig. 2), with some LAs having relatively higher rates of home deaths prior to the pandemic (e.g. Highland) than others (e.g. Scottish Borders). The increase in 2020 was nearly universal with only South Ayrshire, a largely rural region of around 112 000 people, reporting a small decrease in the proportion of home deaths in 2020 followed by a 2021 increase.

## Discussion/conclusions

### Main findings

The home death increase in Scotland since the COVID-19 pandemic continues as of week 25 of 2023, while hospital and care home deaths have returned to pre-pandemic levels and pandemic-related deaths and hospitalizations have decreased. Home deaths increased in all LAs in the 2020–2021 period despite their pre-pandemic variation. Deaths at home had already been increasing prior to the pandemic, but the pandemic increase exceeded the pre-pandemic trend.

### What is already known on this topic

The sustained increase in home deaths during the first 12 months of the pandemic was similar in Scotland to the rest of the UK.^2^ End-of-life care has marked implications for services as well as for unpaid carers, and the increase in home deaths likely affected resourcing. Little is known about the impact of increased deaths at home on end-of-life care, unmet needs or the quality of death during the pandemic period. A modelling attempt in England^11^ estimated additional staff and resource needs for out-of-hospital deaths due to COVID-19, but we are not aware of any estimates for (majority non-Covid) home deaths.

### What this study adds

Assuming a linear trend of home death increases from 2015–2019, the levels of pandemic home deaths would be reached 12 years later; assuming a logistic trend, the pandemic proportion of home deaths would be reached 5 years later. The observed figures of home deaths during the pandemic also exceeded those produced by a 2019 (pre-pandemic) forecasting exercise using Scottish data for 2004–2016, which estimated at most 19 331 deaths at home by 2040, emphasizing the unprecedented scale of the increase beyond that expected from population aging trends.^4^

### Limitations of this study

Publicly available data only describe the point at which death occurred, but not what happened in the final year of life. Because the NRS combines home and other non-institutional settings into a single place of death, the degree to which these figures represent home deaths is uncertain and will include some deaths in public spaces such as those caused by, e.g. traffic accidents, though with the reduction of deadly traffic accidents during the pandemic,^12^^,^^13^ these are unlikely to represent a substantial proportion. Administrative data can only go so far in explaining deaths at home, with questions of quality of care and quality of death requiring qualitative approaches to investigate.

## Supplementary Material

deaths_at_home_short_paper_feb23_supplementary_v1_fdad156

## Data Availability

Weekly deaths data published by NRS are available at https://www.nrscotland.gov.uk/statistics-and-data/statistics/statistics-by-theme/vital-events/general-publications/weekly-and-monthly-data-on-births-and-deaths/deaths-involving-coronavirus-covid-19-in-scotland, under the Open Government Licence (https://www.nationalarchives.gov.uk/doc/open-government-licence/version/3/).

## Notes

**Savinc J,** Research Fellow in Health Data Science

**Atherton I M,** Senior Lecturer, Co-Director/Research Lead
