# Supplementary material for: Have increased deaths at home during the pandemic returned to pre-pandemic levels? An analysis of publicly available Scottish death registrations
Source: J Public Health (Oxf). 2023 Aug 18;45(4):e664–7. doi: 10.1093/pubmed/fdad156 (PMC10687594; doi:10.1093/pubmed/fdad156)
Supplement: deaths_at_home_short_paper_feb23_supplementary_v1_fdad156 [file deaths_at_home_short_paper_feb23_supplementary_v1_fdad156.docx]

Title: Have increased deaths at home during the pandemic returned to pre-pandemic levels? An analysis of publicly available Scottish death registrations

Supplementary file

Code used in the analysis is available on <https://github.com/jsavinc/covid_nrs_place_of_death>.

# Home deaths increased disproportionately to hospital and care home deaths: annual proportions of deaths by place, 2015-2023

Supplementary Figure 1: Annual number of deaths by place in Scotland & relative proportion of deaths by place, 2015-2023.


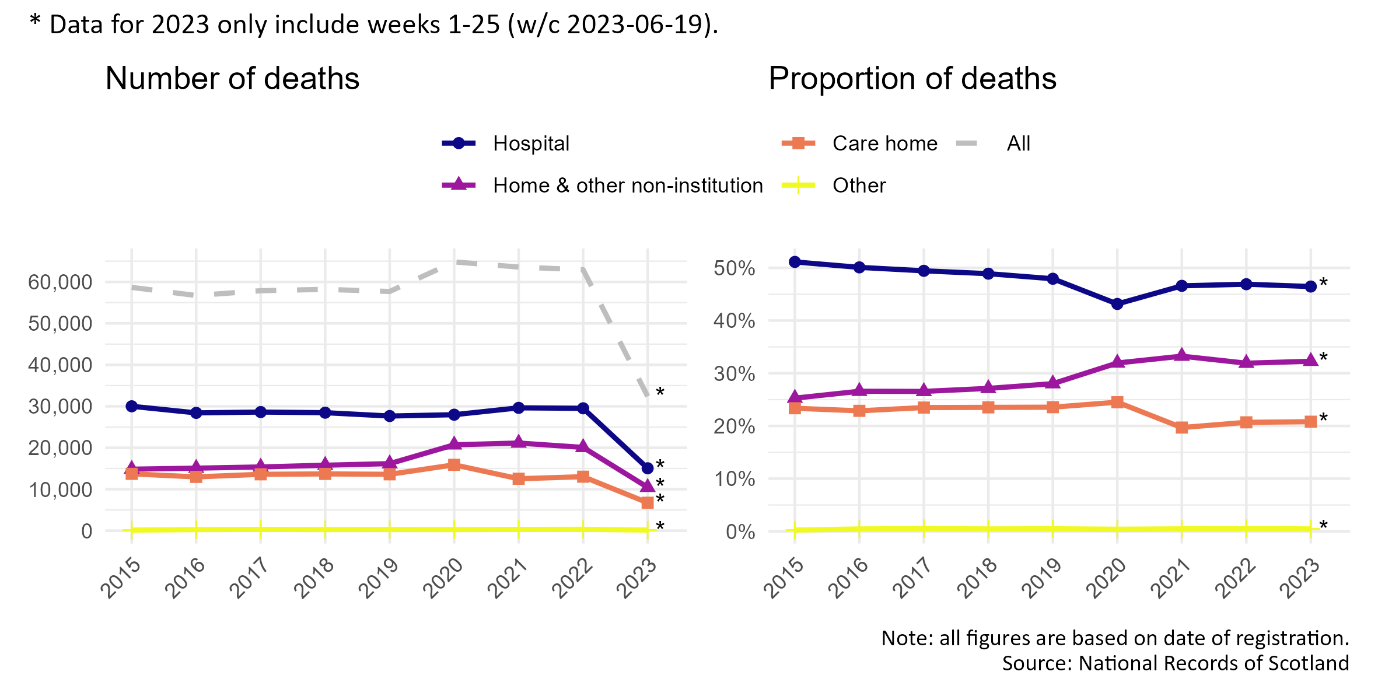


The absolute number of deaths in hospital in 2020 remained very similar to 2019 levels, whereas deaths at home and care homes increased; this is reflected in the relative increase in proportion for home deaths between 2019 and 2020 and the relative decrease of hospital deaths, whilst the proportion of care home deaths increased relatively less. Note that the proportion of hospital deaths increased again between 2020 and 2021 but to a level below 2015-2019 levels; conversely, the proportion of care home deaths decreased to below 2015-2019 levels, whereas the proportion of home deaths increased even further.

| Year | Place of death | Number of deaths | Proportion of total |
| --- | --- | --- | --- |
| 2015 | Hospital | 30000 | 51.1% |
|  | Home & other non-institution | 14831 | 25.3% |
|  | Care home | 13706 | 23.4% |
|  | Other | 140 | 0.2% |
|  | All | 58677 | 100.0% |
| 2016 | Hospital | 28412 | 50.1% |
|  | Home & other non-institution | 15077 | 26.6% |
|  | Care home | 12972 | 22.9% |
|  | Other | 257 | 0.5% |
|  | All | 56718 | 100.0% |
| 2017 | Hospital | 28610 | 49.4% |
|  | Home & other non-institution | 15367 | 26.6% |
|  | Care home | 13594 | 23.5% |
|  | Other | 301 | 0.5% |
|  | All | 57872 | 100.0% |
| 2018 | Hospital | 28458 | 48.9% |
|  | Home & other non-institution | 15787 | 27.1% |
|  | Care home | 13692 | 23.5% |
|  | Other | 265 | 0.5% |
|  | All | 58202 | 100.0% |
| 2019 | Hospital | 27652 | 47.9% |
|  | Home & other non-institution | 16166 | 28.0% |
|  | Care home | 13592 | 23.6% |
|  | Other | 281 | 0.5% |
|  | All | 57691 | 100.0% |
| 2020 | Hospital | 27970 | 43.1% |
|  | Home & other non-institution | 20708 | 31.9% |
|  | Care home | 15886 | 24.5% |
|  | Other | 259 | 0.4% |
|  | All | 64823 | 100.0% |
| 2021 | Hospital | 29624 | 46.6% |
|  | Home & other non-institution | 21139 | 33.2% |
|  | Care home | 12524 | 19.7% |
|  | Other | 299 | 0.5% |
|  | All | 63586 | 100.0% |
| 2022 | Hospital | 29513 | 46.9% |
|  | Home & other non-institution | 20081 | 31.9% |
|  | Care home | 13019 | 20.7% |
|  | Other | 320 | 0.5% |
|  | All | 62933 | 100.0% |
| 2023 | Hospital | 15051 | 46.5% |
|  | Home & other non-institution | 10457 | 32.3% |
|  | Care home | 6741 | 20.8% |
|  | Other | 150 | 0.5% |
|  | All | 32399 | 100.0% |

Supplementary Table 1: Number & proportion of deaths by place, by year. Data for 2023 include weeks 1-25.

# Regression models of home deaths 2015-2019

Two regression models were computed: a linear and a logistic regression model, to estimate the growth of absolute number of home deaths, and the proportion of home deaths, respectively. The aim of both was to compute a simple projection of deaths at home from 2015-2019 onwards in order to estimate at what point the number and proportion of home deaths observed during the pandemic would have been observed if the pre-pandemic trend had continued. This was done to illustrate the scale of the increase rather than to provide a precise estimate, hence the simplicity of the models and the decision to not include any covariates besides time. Seasonality was not included I the model though it was captured by the annual figures.

The linear regression model used the annual absolute number of home deaths as the dependent variable and the year (number of years since 2015) as the explanatory variable. The logistic regression used the annual proportion of home deaths (i.e. the odds of home deaths vs non-home deaths) as the dependent variable and the year (number of years since 2015) as the explanatory variable.

Data for 2023 were available for weeks 1-26. To include 2023 data in the estimates, data for the entire year were imputed: the available number of deaths at home and total deaths (weeks 1-25) were divided by the mean proportion of annual deaths represented by the equivalent weeks in 2015-2019. The average proportion of home deaths in weeks 1-25 in 2015-2019 was .488 and the average proportion of all deaths was .495).

According to the linear model (see Supplementary Table 3), the equivalent absolute number of home deaths observed in 2020, N=20,708, would be observed in the year 2032, the number observed in 2021, N=21,139, in the year 2033, the number observed in 2022, N=20,081, in the year 2030, and the imputed number for 2023, approximately N=21,411, in the year 2034. According to the logistic model, the equivalent proportion of home deaths observed in 2020, .319, would be observed in the year 2025, the proportion observed in 2021, .332, in the year 2027, the proportion observed in 2022, in the year 2025, and the proportion observed in weeks 1-25 of 2023, .323, in the year 2025.

Supplementary Table 2 contains the estimated annual growth in absolute number of home deaths (linear model) and in proportion of home deaths.

| Model | Term | Estimate | SE | Statistic | p |
| --- | --- | --- | --- | --- | --- |
| Linear model, absolute number of home deaths | (Intercept) | 14769.6 | 50.72908 | 291.1466 | 8.94E-08 |
|  | years_since_2015 | 338 | 20.71006 | 16.32057 | 5.01E-04 |
| Logistic model, proportion of home deaths | (Intercept) | 0.342421 | 0.00734 | -146.001 | 0 |
|  | years_since_2015 | 1.031391 | 0.002968 | 10.41297 | 2.16E-25 |

Supplementary Table 2: Regression coefficients for the linear model of absolute home deaths and logistic model of proportion of home deaths. SE: Standard error. Note that estimates for logistic model coefficients are exponentiated.

Supplementary Table 2 contains the actual home deaths, proportions of home deaths, extrapolated annual home and total deaths in 2023, and the estimated year in which the actual absolute number of home deaths and proportions of home deaths in 2020-2023 would have been observed if the modelled pre-pandemic trends continued.

| Year | Home deaths | All deaths | Proportion home deaths | Predicted year (linear model) | Predicted year (logistic model) |
| --- | --- | --- | --- | --- | --- |
| 2015 | 14831 | 58677 | 0.253 |  |  |
| 2016 | 15077 | 56718 | 0.266 |  |  |
| 2017 | 15367 | 57872 | 0.266 |  |  |
| 2018 | 15787 | 58202 | 0.271 |  |  |
| 2019 | 16166 | 57691 | 0.280 |  |  |
| 2020 | 20708 | 64823 | 0.319 | 2032.6 | 2025.2 |
| 2021 | 21139 | 63586 | 0.332 | 2033.8 | 2027.1 |
| 2022 | 20081 | 62933 | 0.319 | 2030.7 | 2025.2 |
| 2023 (weeks 1-25) | 10457 | 32399 | 0.323 |  | 2025.7 |
| 2023  (imputed) | 21411 | 65489 |  | 2034.6 |  |

Supplementary Table 3: Absolute number of home deaths and deaths in all places and proportion of home deaths 2015-2023, including predicted year using linear (based on absolute number of home deaths) and logistic (based on proportion of home deaths) models. Data shown separately for weeks 1-25 of 2023, and for the imputed entire year of 2023. The proportion of home deaths for weeks 1-25 of 2023 was used to predict year when the equivalent proportion would be observed.
